# 

**Published:** 1847-04

**Authors:** 


### THE BRITISH AND FOREIGN MEDICAL REVIEW.

NO. XLVI.—APRIL, 1847.

### PART FIRST.—Analytical and Critical Reviews.

PAGE

1. COPLAND, KENNEDY, LORIMER, HOLLAND, on the Causes and Propagation of Asiatic Cholera . . . . . 313
2. M. CAZENAVE on Syphilitic Eruptions of the Skin . . . 345
3. Dr. MORAND on Medicine and Surgery . . . . 372
4. Professor MATTEUCCI on the Physical Phenomena of Living Beings . . 377
5. Medico-Chirurgical Transactions . . . . . . 410
6. Guy's Hospital Reports . . . . . . . 416
7. Dr. BONE on Yellow Fever, &c. . . . . . . 426
8. Dr. GELLERSTEDT on Tubercular Phthisis . . . . . 429
9. Dr. MOUAT on the Bengal Medical Returns . . . . 440
10. Dr. WATTMANN on the Entrance of Air into the Veins, &c. . . 443
11. Dr. MACKNESS on the Moral Aspects of Medical Life . . . 467
12. Professor OWEN on the Comparative Anatomy and Physiology of the Vertebrate Animals . . . . . . . . . 472
13. LIEBIG and THOMSON on the Food of Animals . . . . 492
14. REMAK'S Diagnostic and Pathogenetic Investigations . . . 503
15. Mr. COOPER on the Sight . . . . . . 510
16. Dr. GLOVER on Scrofula . . . . . . 513
17. SUSRUTA and WISE on Ancient Indian Medicine . . . 521
18. Professor MARX'S Tribute to Stieglitz . . . . 544
19. On Etherization, or the Inhalation of the Vapour of Ether . . 547

### PART SECOND.—Bibliographical Notices.

1. Dr. CHILD on Indigestion . . . . . . . 577
2. Dr. SEARLE'S 'Why and Wherefore' . . . . . . 579
3. Mr. SMEE on the Potato Plant . . . . . . 581
4. Mr. SWAN on the Sympathetic Nerve . . . . . . 582
5. Dr. TURNER'S Elements of Chemistry . . . . . . 584

### PART THIRD.—Natural History and Treatment of Diseases.

1. Dr. MACKENZIE on the Natural Cure of Diseases . . . 585
2. Dr. COMBE on the Observation of Nature in the Treatment of Disease . 592
3. Dr. SYMONDS on the Powers of Nature in the Cure of Diseases . . 596
4. Dr. WARE on the Natural History and Treatment of Delirium Tremens . 603
5. Dr. BALFOUR on the Vienna School and on Homœopathy . . 607

The London and Provincial Medical Directory . . . . . 612

Note from Dr. ASHWELL . . . . . . . ib.

BOOKS RECEIVED FOR REVIEW . . . . . . 613

INDEX, TITLE, &c.

No. XLVII WILL BE PUBLISHED ON THE 1st OF JULY, 1847.

COMMUNICATIONS, JOURNALS, *and* BOOKS FOR REVIEW, *to be forwarded (Carriage paid) to the care of* MR. JOHN CHURCHILL, PRINCES STREET, SOHO; *or direct to the* EDITOR, 12, OLD BURLINGTON STREET, LONDON.

